# Hepatitis C virus core protein induces apoptosis-like caspase independent cell death

**DOI:** 10.1186/1743-422X-6-213

**Published:** 2009-12-01

**Authors:** Christoph P Berg, Stephan F Schlosser, Dorothee KH Neukirchen, Costa Papadakis, Michael Gregor, Sebastian Wesselborg, Gerburg M Stein

**Affiliations:** 1 Department of Internal Medicine I, Medical Clinic, University of Tübingen, Germany

## Abstract

**Background:**

Hepatitis C virus (HCV) associated liver diseases may be related to apoptotic processes. Thus, we investigated the role of different HCV proteins in apoptosis induction as well as their potency to interact with different apoptosis inducing agents.

**Methods and Results:**

The use of a tightly adjustable tetracycline (Tet)-dependent HCV protein expression cell system with the founder osteosarcoma cell line U-2 OS allowed switch-off and on of the endogenous production of HCV proteins. Analyzed were cell lines expressing the HCV polyprotein, the *core *protein, protein complexes of the *core*, envelope proteins *E1*, *E2 *and *p7*, and non-structural proteins *NS3 *and *NS4A*, *NS4B *or *NS5A *and *NS5B*. Apoptosis was measured mainly by the detection of hypodiploid apoptotic nuclei in the absence or presence of mitomycin C, etoposide, TRAIL and an agonistic anti-CD95 antibody. To further characterize cell death induction, a variety of different methods like fluorescence microscopy, TUNEL (terminal deoxynucleotidyl transferase (TdT)-catalyzed deoxyuridinephosphate (dUTP)-nick end labeling) assay, Annexin V staining, Western blot and caspase activation assays were included into our analysis.

Two cell lines expressing the *core *protein but not the total polyprotein exerted a strong apoptotic effect, while the other cell lines did not induce any or only a slight effect by measuring the hypodiploid nuclei. Cell death induction was caspase-independent since it could not be blocked by zVAD-fmk. Moreover, caspase activity was absent in Western blot analysis and fluorometric assays while typical apoptosis-associated morphological features like the membrane blebbing and nuclei condensation and fragmentation could be clearly observed by microscopy. None of the HCV proteins influenced the apoptotic effect mediated via the mitochondrial apoptosis pathway while only the *core *protein enhanced death-receptor-mediated apoptosis.

**Conclusion:**

Our data showed a caspase-independent apoptosis-like effect of the *core *protein, which seems to be inhibited in the presence of further HCV proteins like the non structural (NS) proteins. This observation could be of relevance for the viral spread since induction of an apoptosis-like cell death by the core protein may have some impact on the release of the HCV particles from the host cell.

## Background

Hepatitis C virus (HCV) infection represents one of the most important factors for the generation of chronic hepatitis, liver cirrhosis and hepatocellular carcinoma [1-3]. Since the identification of the virus in 1989 [4], an abundance of investigations had contributed to decipher the molecules and mechanisms involved in the pathogenesis of the disease. However, the properties and signaling mechanisms of the HCV proteins encoded by the viral RNA are still not completely understood. It has been reported that induction of apoptosis is of great importance for the pathogenesis, and two major problems of HCV infection may be related to apoptosis, i.e. the viral persistence and the direct or indirect destruction of liver cells. Therefore, the study of host-virus interactions, especially the influence on the regulation of apoptotic processes by the different viral proteins is poorly defined but may help explain these problems. Thus, if viral proteins inhibit host cell apoptosis this effect may contribute to the viral persistence since the virus escapes the immunological attack. On the other hand, if viral proteins induce apoptosis in the host cell, this may be an important factor for liver cell destruction.

From a variety of viruses it is well known that they employ different apoptotic signaling components in the host cell for inhibition or activation of the endogenous suicide program. Thus, some viruses are able to induce apoptosis of the host cell *via *their newly synthesized virus-specific proteins [5-7], while virus-specific proteins from other viruses act as anti-apoptotic agents [8-12]. Similar observations were made for the hepatitis C virus, showing that the virus may destroy hepatocytes by induction of apoptosis. In addition, CD4+ and CD8+ T-cells are involved in the inflammatory process as well as the destruction of these cells by directly inducing cytotoxic effects *via *apoptosis or indirectly by secretion of different cytokines [13]. On the other hand, inhibition of apoptotic processes creates a privileged milieu for the replication and propagation of HCV [14]. Furthermore, inhibition of apoptosis may play a major role in the generation of hepatocellular carcinoma [15,16].

In the past, the apoptotic and anti-apoptotic effects of different HCV proteins have been intensively studied. However, conflicting data were generated depending on the experimental conditions, i.e. methods and cell lines used. E.g. in transfected HepG2, Jurkat T or COS-7 cells endogenously expressing the *core *protein or the full length HCV polyprotein, induction of apoptosis was observed [17-19]. In contrast, stably transfected B cells expressing the *core *protein did not exert any apoptotic effect [20]. In addition, studying the effect of 'non-*core' *HCV proteins conflicting results have also been found with respect to their potency to stimulate apoptotic processes [21-23].

A similar situation could be observed studying the influence of the HCV on the extrinsic receptor-mediated and intrinsic mitochondrial apoptosis pathway. Thus, a slight inhibition of the death receptor-mediated apoptosis by the endogenously expressed core protein was described [24], while other authors found an increase of the Fas-mediated apoptosis by the transfected cells expressing the core protein using the same founder cell line [25,26]. These data demonstrate that the experimental settings like the use of different vectors, different kinetics, cell cultures, or detection methods may influence the results and render a generalized statement more difficult. Thus, the objective of our study was to investigate the effect of a spectrum of HCV proteins and protein complexes in a tightly adjustable HCV protein expression cell system which allowed switch off and on of the endogenous production of HCV proteins [27-31]. Using this tetracycline-regulated (Tet-off) system we studied the influence of different HCV proteins on apoptosis induction and on the receptor-mediated and mitochondrial pathway of apoptosis stimulated by different agents.

## Methods

### Tetracycline-regulated cell lines

All tetracycline-regulated cell lines (Table 1) were a kind gift from Darius Moradpour, Division of Gastroenterology and Hepatology, Centre Hospitalier Universitaire Vaudois, Lausanne, Switzerland, and were generated using the constitutively tetracycline-controlled transactivator (tTA)-expressing U-2 OS osteosarcoma cell line (ATCC HTB-96) as described [27-33] (Moradpour unpublished). All cell lines were maintained in culture in Dulbecco's MEM (invitrogen Life Technologies, Karlsruhe, Germany) supplemented with 10% heat-inactivated fetal calf serum (PAA laboratories, Cölbe, Germany), 500 μg/ml Geneticin (G418; invitrogen), Glutamax 2 mM (invitrogen), 50 units/ml penicillin (invitrogen), 5 μg/ml streptomycin (invitrogen), 1 μg/ml puromycin (Sigma, Deisenhofen, Germany) and 1 μg/ml tetracycline (Tet, Sigma) [29,30]. Cells were grown at 37°C in a 5% CO2 atmosphere in the log phase. Adding tetracycline to the different cell lines blocks the expression of the HCV proteins. On the other hand cells were washed twice with PBS (invitrogen) and incubated in medium without tetracycline to induce HCV protein expression.

**Table 1:** HCV-proteins expressed in the different cell lines

cell lines	expressed HCV-proteins	clones	**Ref**.
UHCV	ORF	UHCV-32	[30]
UC	p21 (core-protein)	UCcon-39	Moradpour, unpublished
UCp7	p21-p7 (core-protein-E1-E2-p7)	UCp7con-11.17	Moradpour, unpublished
UNS3-4A	NS3, NS4A	UNS3-4A-24	[31]
UNS4B	NS4B	UNS4Bcon-4	[27]
UNS5A	NS5A	UNS5A	[32]
UNS5B	p68 (NS5B)	UNS5Bcon-5	[33]

* E: envelope, NS: non structural protein, ORF: open reading frame

### Apoptosis and cell viability assays

Apoptosis was measured by flow cytometry using the Nicoletti method to detect the leakage of fragmented DNA from apoptotic nuclei [34,35]. Briefly, the different cell lines were grown in the presence or absence of tetracycline and/or in the presence or absence of different apoptosis inducing agents for the indicated times at a concentration of 1 × 10^5^/ml in 96-well (200 μl) or 24-well plates (1 ml) and cultured for 48 h if not stated otherwise. In some assays, cells were pre-incubated with the broad-range caspase inhibitor benzyloxycarbonyl-Val-Ala-Asp-fluoromethylketone (zVAD-fmk; 100 μM; Bachem, Heidelberg, Germany) for 24 h before the apoptotic stimuli were added for another 24 h. Apoptosis was induced exogenously by TRAIL (TNF-receptor-associated apoptosis inducing ligand; 40 ng/ml; R&D systems, Heidelberg, Germany), anti-CD95 antibody (100 ng/ml; CH11; upstate/Biomol, Hamburg, Germany), etoposide (400 ng/ml; Sigma), or mitomycin C (50 μg/ml; Medac, Wedel, Germany).

In further experiments a variety of protease inhibitors of signal transduction were added to the cultures at day 0: leupeptin (100 μM; Böhringer Mannheim, Mannheim, Germany), pepstatin A (50 μM; Böhringer Mannheim), cathepsin B inhibitor Ca-074 (30 μM; Calbiochem, Bad Soden, Germany), calpain inhibitor II (N-Ac-L-Leuc-L-Leucyl-L-methioninal; 10 μg/ml; Sigma), pefabloc (0.3 mM; Roche, Mannheim, Germany), oligomycin (10 μM; Calbiochem), LY294002 (20 μM; inhibitor of PI3 kinase; Cell signaling, Beverley, USA), and ROCK inhibitor Y-27632 (100 μM; Calbiochem).

At the end of the incubation period, cells were collected and lysed for 10 min in 100 μl of hypotonic buffer (0.1% sodium citrate, 0.1% Triton X-100, 50 μg/ml propidium iodide (PI)). Apoptotic nuclei were detected by flow cytometry (FACSCalibur; BD, Heidelberg, Germany) using the CellQuest analysis software. Nuclei to the left of the 2 N peak containing hypodiploid DNA were considered apoptotic [35,36]. Analyses were performed in triplicates and mean and standard deviation are provided in the Figures.

Apoptosis was also detected by Annexin V/PI staining as reported after trypsinization of the cells after a 48 h culture period [37].

For the determination of cell viability using the methyltetrazolium salt (MTS) test, 1 × 10^5 ^cells/ml were incubated in the presence or absence of Tet and the apoptotic stimuli for the times indicated. Subsequently, MTS (450 μg/ml; 3-(4,5-Dimethylthiazol-2-yl)-2,5-diphenyltetrazoliumbromid, Sigma) was added to the cells for 4 h at 37°C. Resulting formazan crystals were dissolved in 4% SDS and measured at 550 nm. Analyses were performed in triplicates and mean and standard deviation are provided in the Figure.

### Western blot analyses

For the detection of HCV and apoptosis-related proteins, Western blot analyses were performed following the method described previously with slight modifications [35,36,38,39]. As primary antibodies mouse monoclonal antibodies (moAbs) directed against caspase-8 (1:10 dilution of a hybridoma supernatant; Cell Diagnostica, Germany), caspase-3 (1 μg/ml; Transduction Laboratory, Heidelberg, Germany), PARP (poly-ADP-ribose polymerase; 1:2,000; Alexis, Hiddenhausen, Germany), the *core *protein and the NS3 protein (1:1,000) [31,40] were used. HRPO-conjugated secondary antibodies to mouse IgG (1:4,000; Biorad, Munich, Germany) allowed the use of the ECL plus technique (Amersham-Buchler, Braunschweig, Germany) to visualize the antigens after extensive washing.

### Fluorometric assay of caspase activity

Analyses of the caspase activity using cytosolic cell extracts of 2 × 10^4 ^cells were performed as described [39].

### Microscopy

To study morphological alterations of the cell lines, microscopic analysis were performed. Therefore, 2 × 10^4 ^cells/well were cultured in chamber slides (Lab Tek, Brand Products, Germany) in the presence or absence of Tet and zVAD-fmk (100 μM) for 24 h. Afterwards, mitomycin C (50 μg/ml), TRAIL (40 ng/ml), or anti-CD95 antibody (100 ng/ml) were added for another 24 h. Nuclei were stained with the cell permeable dye Höchst 33342 (2 μg/ml; Sigma) for 10 min at 37°C and investigated by fluorescence microscopy using the Axiovert 135 microscope (Zeiss, Jena, Germany). Analyses were performed in triplicates.

### TUNEL

To evaluate the induction of DNA-fragmentation by the terminal deoxynucleotidyl transferase (TdT)-catalyzed deoxyuridinephosphate (dUTP)-nick end labeling (TUNEL) assay, 5 × 10^5 ^cells/ml were cultured for 24 h in the presence and absence of Tet and zVAD-fmk (100 μM) before mitomycin C (50 μg/ml) or TRAIL (40 ng/ml) were added for another 24 h. DNA-fragments were detected using the MEBSTAIN Apoptosis kit Direct (Coulter-Immunotech, Krefeld, Germany) following the instructions of the manufacturer as described [41,42].

## Results

### 1. Induction of hypodiploid nuclei by the HCV core protein

**Figure 1:**
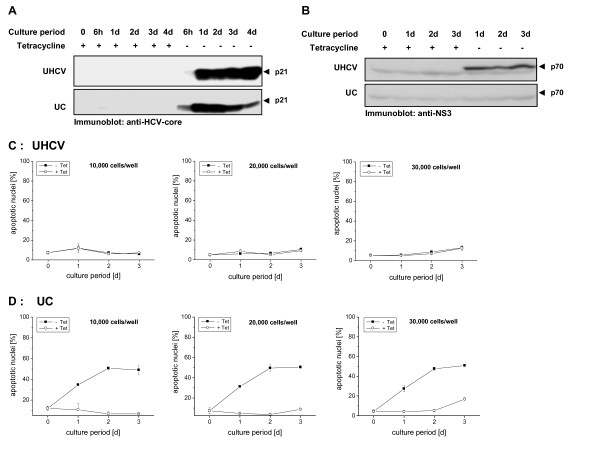
**Expression of different HCV-proteins in the UHCV and UC cells (A, B) and their induction of apoptotic nuclei (C, D): 2 × 10^6 ^cells (A, B) or 1 × 10^4^, 2 × 10^4^, and 3 × 10^4 ^cells/well (C, D) of each cell line were cultured for the indicated time in the presence or absence of tetracycline (Tet) to induce HCV-specific protein expression**. A, B: Cellular proteins were resolved by SDS-PAGE and HCV proteins were detected by immunoblotting with an antiserum generated against the *core *(A) or NS3 protein (B) of HCV. C, D: Induction of apoptosis was assessed by flow cytometric analysis of propidium iodide staining of hypodiploid apoptotic nuclei. The mean values and standard deviation of triplicate cultures are shown.

In order to compare the potency of the different HCV proteins to induce apoptosis, we first studied the expression of the proteins produced by the UHCV cell line coding for the ORF and the UC cell line coding for the *core *protein. Figure 1A demonstrates by Western blot analysis in a kinetic study that in the absence of tetracycline (Tet) the *core *protein is strongly synthesized in both cell lines, while the NS3 protein, exemplary shown for the expression of further HCV proteins, is present only in the Tet-off UHCV but not the UC cell culture (Figure 1B). Thus, within the UHCV cell line the polyprotein is cleaved to release the single HCV proteins.

To study the effects of the *core *protein and the whole HCV proteins on apoptosis induction, we analyzed the typical apoptosis-associated leakage of fragmented DNA from apoptotic nuclei by the Nicoletti method using flow cytometry. As shown in Figure 1C in kinetic studies, there was no apoptotic effect detectable in the polyprotein expressing UHCV cell line, independent from the cell number seeded. In contrast, the *core *protein expressed in the UC cell line in the absence of Tet led to a strong leakage of fragmented DNA already after one day (Figure 1D). The typical apoptotic effect depended on the expression level of the *core *protein and not on the cell density employed. Thus, testing two high and two low expression cell lines from the UHCV and the UC cells, DNA fragmentation was induced only in the UC cell line with an elevated expression of the *core *protein (data not shown).

### 2. Cell death could not be induced by further HCV proteins

**Figure 2:**
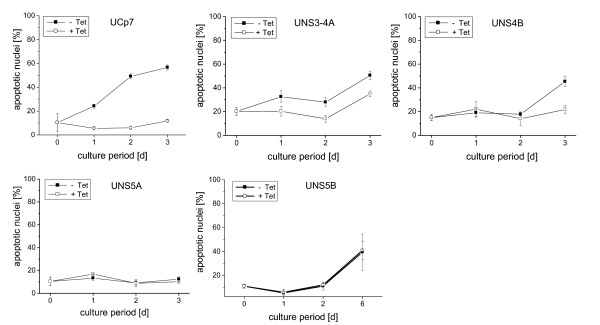
**Induction of apoptosis in different HCV-protein expressing cell lines: 2 × 10^4 ^cells of the different cell lines UCP7, UNS3-4A, UNS4B, UNS5A, and UNS5B were cultured for the indicated times in the presence or absence of Tet to induce specific protein expression**. Induction of apoptosis was assessed by propidium iodide staining of hypodiploid apoptotic nuclei and flow cytometry. The mean values of triplicate cultures and standard deviation are shown.

Next, we addressed the question, whether further HCV proteins expressed in our test system also exert cell death inducing properties. Therefore we tested a variety of cell lines expressing different single HCV proteins or protein groups by flow cytometry [27,31,32,43]. However, a strong effect on the generation of hypodiploid nuclei could only be observed in the cell line UCp7 expressing the *core*, E1, E2 and p7 protein, whereas the other cell lines did not exert any or only a slight (NS3-4A and NS4B proteins) effect (Figure 2). For the NS3-4A cells the increase of apoptotic cells after 3 days was independent from the NS3-4A protein since the difference in the rate of apoptotic nuclei between the induced and the non-induced cells was constant from day 1 to day 3. Possibly, this is a problem of the position of the insert coding for the HCV protein in this cell line.

Since we did not observe any difference in the rate of apoptotic nuclei in the absence of Tet in the NS5B cells after 2 days, we further studied the activity after a quite longer period, i.e. 6 days. However, we only found an unspecific increase, most likely due to the consumption of nutrients in the cell culture medium.

### 3. Apoptotic features induced by the HCV core protein

**Figure 3:**
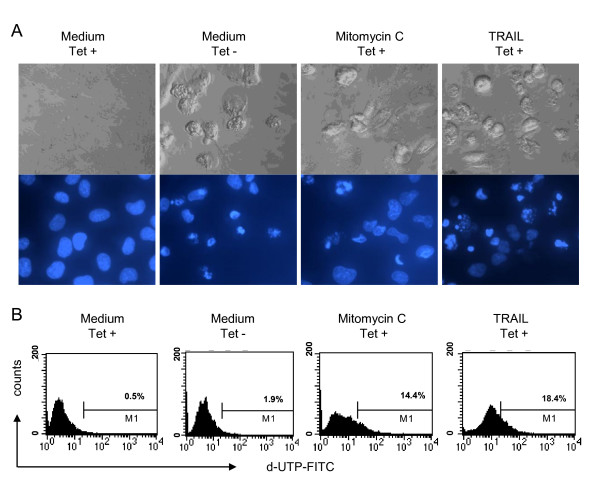
**Different features of apoptosis induced by the HCV-*core *protein in UC cells cultured in the presence or absence of tetracycline to induce specific protein expression**. A: membrane blebbing and nuclear condensation was visualized in 2 × 10^4 ^UC cells cultured for 48 h. Apoptotic stimuli were added during the last 24 h: mitomycin C (50 μg/ml), and TRAIL (40 ng/ml). Nuclei were stained with the cell permeable dye Höchst 33342 and cells were applied to phase contrast and fluorescence microscopy (magnification: 320 ×). B: Detection of apoptosis by the TUNEL assay. 5 × 10^5 ^cells/ml were cultured for 48 h and mitomycin C (50 μg/ml) and TRAIL (40 ng/ml) were added during the last 24 h and served as positive control. TUNEL assay was measured by flow cytometry.

In order to characterize more precisely cell death induction by the *core *protein we analyzed the reactivity of the UC cell line by different methods. Thus, we observed by phase contrast and fluorescence microscopy (magnification 320×) that the *core *protein induced typical morphological features of apoptosis: Similar to mitomycin C and TRAIL, which served as positive controls, the *core *protein stimulated apoptotic blebs on the cell surface (Figure 3A). In addition, nuclei were condensed and fragmented in these cells as evidenced by the staining pattern with the Hoechst dye 33342 (Figure 3A). However, in the TUNEL assay detected by flow cytometry there was only a slight increase in the amount of fragmented nuclei which were accessible for the TdT in response to the core protein as compared to the positive controls (Figure 3B).

### 4. Influence of the HCV proteins on death receptor-mediated and mitochondrial apoptosis pathways

**Figure 4:**
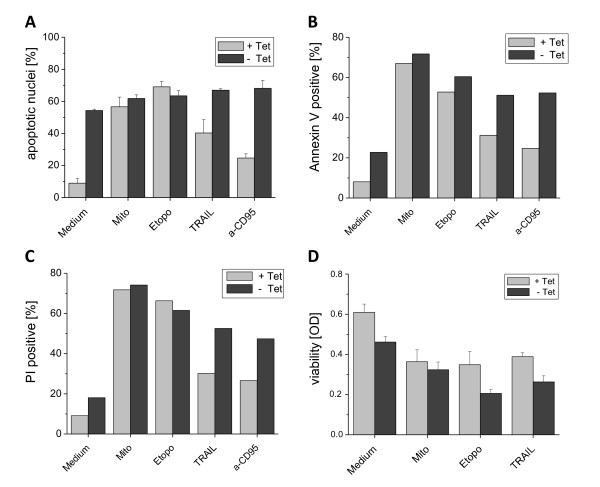
**Influence of HCV-*core *protein on the receptor-mediated and the mitochondrial apoptosis pathway studied in different assays: 1 × 10^5 ^UC cells/ml were cultured for 48 h in the presence or absence of Tet to induce specific protein expression**. Apoptotic stimuli were added during the last 24 h: mitomycin C (50 μg/ml), etoposide (400 ng/ml), TRAIL (40 ng/ml) and anti-CD95 antibody (100 ng/ml). A: Induction of apoptosis was assessed by flow cytometric analysis of propidium iodide staining of hypodiploid apoptotic nuclei. The mean values and standard deviation of triplicate cultures are shown. B: Apoptosis was visualized by the externalization of PS which was stained with Annexin V and C: viability of the cells was measured by staining of the cells with PI and subsequent detection by flow cytometry. Given are means of duplicates. D: Metabolic activity of the UC cells was determined by the MTS test. Optical density was measured in an ELISA reader after incubation of the cells with MTS for 4 h and suspension of crystals. Given are mean and standard deviation of triplicates.

Since in our experiments the major effect was induced by the *core *protein, we focused in our further studies on the UC cell line. To investigate whether the HCV *core *protein exerts an enhancing effect on the activation of the death receptor pathway or the mitochondrial apoptosis pathway we first stimulated the expression of the HCV proteins for 24 h and added a variety of apoptosis inducers to the cell cultures for another 24 h. For stimulation of death receptors we used agonistic anti-CD95 antibodies or the DR4 and DR5 ligand TRAIL and for the activation of the mitochondrial apoptosis pathway we used the anticancer drugs mitomycin C and etoposide, as previously described [39]. As shown in Figure 4A, a costimulatory effect of the core protein expressed by the UC cells on the rate of hypodiploid nuclei measured by flow cytometry could be observed only in the TRAIL and anti-CD95 stimulated cells as compared to the non-core expressing cells.

Figure 4B demonstrates that the *core *protein alone slightly enhanced the phosphatidylserine (PS) externalization and further enhanced the effect of the apoptotic agents acting *via *the receptor-mediated pathway as measured by the staining with Annexin V by flow cytometry. Similar observations were made for the uptake of propidium iodide that measures cell death in general and cannot discriminate between apoptosis and necrosis (Figure 4C). In addition, the viability of the cells expressing the *core *protein was reduced by the *core *protein as evidenced by a diminished formazan crystallization in the MTS test (Figure 4D).

However, analyzing the UHCV, UNS4B, and NS5A cell lines, there was no significant difference in response to the exogenously added apoptotic stimuli between the cells expressing the respective HCV proteins or not (data not shown).

### 5. Cell death induction by the core protein is not caspase-dependent

**Figure 5:**
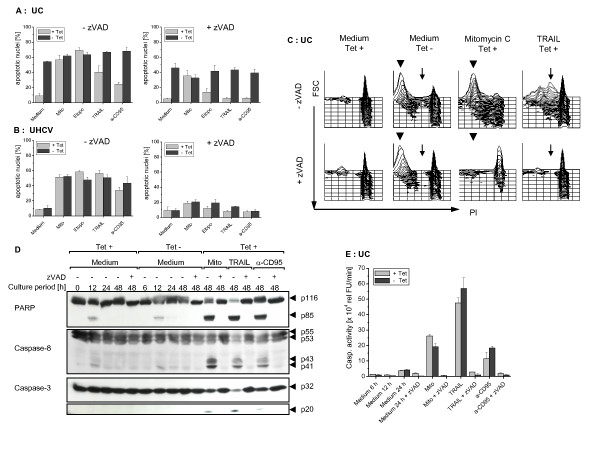
**Influence of the HCV-*core *protein on caspase activation**. 1 × 10^5 ^cells/ml of the UC (A, C, E) and UHCV cell lines (B) or 1 × 10^6 ^UC cells (D) were cultured for the indicated times in the presence or absence of Tet to induce specific protein expression and the broad spectrum caspase inhibitor zVAD (100 μM). The apoptotic stimuli mitomycin C (50 μg/ml), etoposide (400 ng/ml), TRAIL (40 ng/ml), and anti-CD95 antibody (100 ng/ml) were added during the last 24 h of the culture. The broad spectrum caspase inhibitor zVAD was added at day 0 if indicated. A-C: Induction of apoptosis was assessed after 48 h by flow cytometric analysis of propidium iodide staining of hypodiploid apoptotic nuclei. The mean values and standard deviation of triplicate cultures are shown (A+B). D: Cleavage of caspases-3 and -8 as well as of PARP was detected in the cell lysates by Western Blot analysis. E: Detection of the caspase activity in UC cells was performed by *in vitro *cleavage of the fluorogenic substrate DEVD-AMC and was measured by fluorometry at the time indicated. Apoptotic stimuli were added for 24 h. The mean values and standard deviation of triplicate cultures are given.

In order to study whether caspases are involved in the process of cell death induction by the *core *protein, we first stimulated the core expressing UC cell line in the presence or absence of the broad spectrum caspase inhibitor zVAD-fmk. As shown in Figure 5A, the *core *protein induced generation of hypodiploid nuclei was only partially affected by zVAD-fmk, whereas zVAD-fmk clearly inhibited their generation stimulated by mitomycin C, etoposide, TRAIL, and anti-CD95 antibody in the Tet-on cells. In contrast, in the polyprotein expressing UHCV cell line generation and inhibition of apoptotic nuclei using different apoptotic stimuli with or without zVAD-fmk was independent of the Tet-off system (Figure 5B).

Despite the observation that the UC cell line was less sensitive to the receptor-mediated apoptosis pathway, an additional apoptotic effect could be observed by the *core*-protein (Figure 5A). This effect could only partially be inhibited by zVAD-fmk suggesting that a caspase-independent mechanism may be responsible for the *core *protein induced cell death.

Studying in more detail the *core *protein mediated apoptosis it became evident that zVAD-fmk did not inhibit the *core *protein-induced generation of hypodiploid nuclei, in contrast to cell death induction due to Mitomycin C and TRAIL which showed an almost complete inhibition following application of zVAD-fmk (Figure 5C). Interestingly, most hypodiploid nuclei were very small in the core protein expressing cells as compared to the nuclei arising after stimulation with TRAIL. While zVAD-fmk did not inhibit the *core *protein-induced generation of hypodiploid nuclei, it almost completely blocked the small nuclei induced by mitomycin C.

To directly analyze the involvement of caspases in the action of the *core *protein, Western blot analyses were performed confirming that both, caspases-3 and -8, had not been activated since neither caspase cleavage products could be observed, nor did they comprise any activity, as demonstrated by the lack of the cleavage of the caspase substrate PARP (Figure 5D). In contrast, cultivation with the typical apoptotic stimuli mitomycin C, TRAIL or the stimulatory anti-CD95 antibody induced caspase activation that could be inhibited by zVAD-fmk.

In addition, using the fluorogenic substrate DEVD-AMC in a fluorometric assay we could not observe any core protein related caspase activity (Figure 5E). Cell lysates of the Tet regulated *core *expressing UC cell line did not possess any caspase activity, in contrast to the lysates of cells incubated with mitomycin C, TRAIL or the anti-CD95 antibody which showed a typical caspase activity. Similar observations were made with the UHCV cell line (data not shown).

Additional experiments were performed to study whether ICAD (inhibitor of caspase activated DNAse) was cleaved by the *core *protein which in turn may lead to the activation of the endonuclease CAD (caspase activated DNAse). However, we could not observe any cleavage of ICAD by the *core *protein (data not shown) which further confirms a caspase-independent type of DNA cleavage.

### 6. Analysis of the involvement of a variety of protease inhibitors in the apoptosis-like effect of the core protein

**Figure 6:**
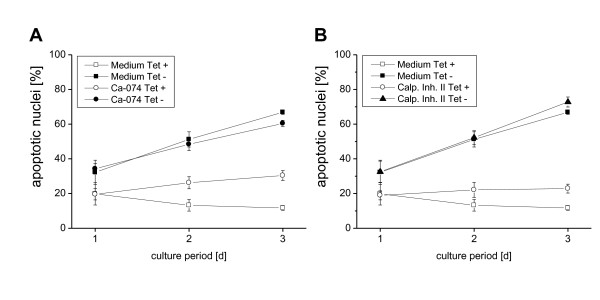
**HCV *core *protein induced apoptosis could not be completely inhibited by a broad spectrum of protease inhibitors**. 1 × 10^5 ^UC cells/ml were cultured for the indicated times (A, B) in the presence or absence of Tet to induce specific protein expression. The protease inhibitors were added at day 0: cathepsin inhibitor Ca-074 30 μM, and calpain inhibitor II 10 μM. Induction of apoptosis was assessed by propidium iodide staining of hypodiploid apoptotic nuclei and flow cytometry. The mean values and standard deviation of triplicate cultures are given.

To study in more detail the mechanisms involved in the apoptosis-like activity of the *core *protein, we tested a variety of broad-spectrum as well as specific protease inhibitors for their ability to block the *core *protein-induced generation of apoptotic nuclei (Figure 6). In these kinetic studies, neither the cathepsin B inhibitor (Figure 6A) nor the calpain inhibitors I (data not shown) and II (Figure 6B) exerted any effect on the *core *protein-induced apoptosis. In addition, none of the other specific and unspecific inhibitors as leupeptin, pepstatin, pefabloc, ROCK inhibitor and oligomycin were able to block the apoptotic effect after 48 h of cell culture, while the inhibitor of the PI3 kinase LY294002 and the calpain inhibitor I were toxic (data not shown).

## Discussion

The objective of our study was to investigate the potency of endogenously expressed HCV proteins on apoptosis induction and to analyze their influence on the death receptor-mediated and the mitochondrial apoptosis pathway. To address these questions, we used a recently established tightly adjustable HCV protein expression cell system which allowed switch off and on of the endogenous production of a broad spectrum of HCV proteins or protein complexes (Tet-off system) [27-31]. Using this system we compared the apoptosis-inducing effects of the different single HVC proteins and protein complexes. This is of major importance since the literature presents conflicting data on that topic. It could be shown that e.g. the receptor-mediated apoptosis was inhibited by the *core *protein [24] while just the opposite effect was obtained by different authors, even if the same cell line was used [25,26]. These data demonstrate that the observed effects strongly depend upon the experimental conditions. To circumvent this problem by using cell lines inducible expressing a broad spectrum of HCV proteins and protein complexes it became evident that the cell lines expressing the *core *protein (i.e. UC and UCp7) showed a strong induction of apoptotic nuclei. The other HCV proteins and protein complexes did not show any effect with the exception of a very slight stimulation by the *NS3-4A *and *NS4B *proteins.

Cell death induction of the *core *protein expressing cells was evidenced by a variety of methods. Thus, typical apoptosis-associated morphological alterations like the loss of the contact to neighboring cells, formation of apoptotic blebs and nuclear condensation could be clearly detected. In addition, a slight externalization of phosphatidylserine as well as a diminished metabolic activity induced by the *core *protein fit to these observations. The best read-out system for the analysis of the apoptotic effect was the visualization of hypodiploid nuclei. Interestingly, these nuclei were very small, similar to those obtained by stimulation with mitomycin C but their generation could not be blocked by the caspase inhibitor zVAD-fmk in contrast to that observed for mitomycin C or TRAIL. In addition, the typical 'DNA-ladder' obtained after internucleosomal cleavage of DNA could not be observed in the UC cell line (data not shown). Moreover, using the less sensitive TUNEL assay we did not find any *core *protein related typical apoptosis-associated DNA fragmentation pattern while mitomycin C and TRAIL were active in this test system. The lack of reactivity of the *core *protein in these two assays is in accordance with the lack of caspase activation since the internucleosomal cleavage of DNA is mainly due to the activity of CAD (caspase-dependent DNase) during apoptosis, which is inhibited by ICAD (inhibitor of CAD) [44,45]. Consistently with the lack of caspase activation, we, in contrast to Sacco *et al*. [46], did not observe alterations of ICAD (data not shown). From all these data it is assumed that the *core *protein stimulated apoptosis-like cell death is mainly caspase-independent. Caspase-independent apoptosis pathways have been described and are now generally accepted [47]. In contrast to our data, Moorman *et al*. and Goh *et al*. observed an activation of caspases-3 and -8 by the *core *protein [19] or the cleavage of PARP [17] which may be strongly influenced by the experimental conditions.

Analyzing the apoptotic effect of the UCp7 cells we cannot completely exclude that it was influenced by the proteins *E1*, *E2 *or *p7*, although it seems reasonable that the *core *protein was responsible for the major effect. However, in this respect two publications may be of relevance showing apoptotic effects of the *E1 *[48] and the *E2 *protein [49]. Thus, further investigations should include cells expressing either protein in the same adjustable system.

In order to better define the mechanisms involved in the apoptosis-like machinery stimulated by the *core *protein, we tried to block the *core *protein induced generation of hypodiploid nuclei by a variety of proteases *via *unspecific and specific inhibitors. However, none of the different protease inhibitors, like the specific inhibitor of cathepsin B (Ca-074) or calpains or the unspecific inhibitors of proteases like leupeptin and pepstatin could block cell death induction. In future experiments further signal transduction cascades like the Akt/PKB and other signaling pathways have to be investigated.

One intriguing finding was that the polyprotein expressing UHCV cells did not exert any apoptotic effect although they clearly expressed the *core *protein. This observation is difficult to explain and may reflect the complexity of the virus-specific reactions. It may be possible that both, the *core *protein associated apoptotic and the possible anti-apoptotic effects of further HCV proteins may act together. Thus, in stably transfected cell lines, Chung *et al*. found an anti-apoptotic effect of the *NS5A *protein while the *core *protein exerted apoptotic potency, similar to our data [50]. In addition, it has been described that different HCV proteins like the *NS3 *and *NS4A *or *NS4A*, *NS4B *and *NS5A *interact [51,52]. Thus, it cannot be excluded that different proteins also bind to the *core *protein or signaling molecules of the *core *protein induced cascade and block its effect. This regulation of apoptosis may be of advantage for the virus in order to circumvent a premature apoptosis before the virus replication and assemblage has finished.

The second objective of our investigations was to study the influence of the HCV proteins on apoptosis induced by exogenous stimuli acting on the mitochondrial (mitomycin C and etoposide) or the death receptor-mediated (TRAIL, agonistic anti-CD95 antibody) apoptosis pathway [35,36,39]. None of the tested cell lines, i.e. the UHCV, UNS4B and UNS5A cell lines exerted a significant stimulatory or inhibitory effect on either apoptosis pathway. In contrast, the UC cell line enhanced the TRAIL and anti-CD95 mediated apoptosis as evidenced by an increase of cell death-related features studied in different test systems.

From the data presented here it appears that the *core *protein did not exert its major effect *via *the receptor mediated pathway by inducing the respective ligands on the neighboring cells. However, we cannot exclude that this mechanism is operative in the *core *protein mediated enhancement of apoptosis induced by death receptor ligands.

Comparing our data on the influence of the HCV proteins on exogenous apoptotic stimuli with the data in the literature our results in part are in accordance with previously published work since some authors did not find an influence of the *core *protein on the receptor-mediated apoptosis pathway [53], an increase [26,54] or an inhibition [24].

In contrast to the available data on the *core *protein, the data on the *NS5A *protein are more uniform demonstrating a rather anti-apoptotic effect for that protein [22,23,55-58].

From the results obtained in our study it is evident that the *core *protein exerted the strongest caspase-independent direct apoptosis-like effect whereas none of the other HCV proteins showed a clear-cut influence on exogenous apoptotic stimuli. In this respect the localization of the mature *core *protein at the outer mitochondrial membrane may be of importance [59]. Although we did not observe the release of cytochrome c in the Tet-off UC cell line (data not shown) an interaction of the *core *protein with other molecules of the apoptotic machinery localized at the mitochondrial membranes may occur. Thus, further investigations are necessary to better characterize the role of the different HCV and host cell proteins in the apoptotic processes. Since some of the proteins interact with each other, more complex systems are needed for a more precise evaluation of the pathology of the disease in order to develop new remedies with an anti-viral effect to HCV.

## Conclusion

In our experiments the non-structural proteins seem to exert an anti-apoptotic effect since in the polyprotein expressing cells no apoptosis-like features could be observed. Thus, it is tempting to speculate that *in vivo *they may inhibit early host cell death while *core *protein stimulated caspase-independent apoptosis-like effect may follow at later stages and, therefore, could be of relevance for the release of the HCV particles from the host cell and the viral spread.

## List of abbreviations

CAD: caspase-dependent DNase; DEVDamc: N-acetyl-Asp-Glu-Val-Asp-aminomethylcoumarin; E: envelope; etopo: etoposide; HCV: hepatitis C virus; ICAD: inhibitor of CAD; mito: mitomycin; MTS: methyltetrazolium salt; NS proteins: non structural proteins, ORF: open reading frame; Tet: tetracycline; TNF: tumor necrosis factor; TRAIL: TNF-related apoptosis inducing ligand; TUNEL: terminal deoxynucleotidyl transferase (TdT)-catalyzed deoxyuridinephosphate (dUTP)-nick end labeling; zVAD: benzyloxycarbonyl-Val-Ala-Asp-fluoromethylketone.

## Competing interests

The authors declare that they have no competing interests.

## Authors' contributions

CPB and SFS contributed equally to this paper and share first authorship. GMS, CPB, DKHN and CP performed research. Also GMS and SW contributed equally to this paper and share senior authorship. GMS, CPB, SFS and SW designed research, analyzed data and wrote the manuscript. MG helped discussing the data. They all read and approved the final manuscript.
